# Protective Mechanism of Edible Food Plants against Alcoholic Liver Disease with Special Mention to Polyphenolic Compounds

**DOI:** 10.3390/nu13051612

**Published:** 2021-05-11

**Authors:** Liang Zhao, Arshad Mehmood, Dongdong Yuan, Muhammad Usman, Mian Anjum Murtaza, Sanabil Yaqoob, Chengtao Wang

**Affiliations:** 1 Beijing Advance Innovation Center for Food Nutrition and Human Health, Beijing Technology and Business University, Beijing 100048, China; liangzhao@btbu.edu.cn (L.Z.); arshadfst@yahoo.com (A.M.); ch.usman1733@gmail.com (M.U.); wangchengtao@th.btbu.edu.cn (C.W.); 2 Beijing Engineering and Technology Research Center of Food Additives, School of Food and Health, Beijing Technology and Business University, Beijing 100048, China; 3 Institute of Food Science and Nutrition, University of Sargodha, Sargodha 40100, Pakistan; anjum.murtaza@uos.edu.pk; 4 Department of Food Science and Technology, University of Central Punjab, Punjab 54590, Pakistan; Sanabil.yaqoob@ucp.edu.pk

**Keywords:** alcoholic liver disease, bioactive compounds, polyphenols, antioxidants, gut microbiota

## Abstract

Alcoholic liver disease (ALD) is one type of liver disease, causing a global healthcare problem and mortality. The liver undergoes tissue damage by chronic alcohol consumption because it is the main site for metabolism of ethanol. Chronic alcohol exposure progresses from alcoholic fatty liver (AFL) to alcoholic steatohepatitis (ASH), which further lead to fibrosis, cirrhosis, and even hepatocellular cancer. Therapeutic interventions to combat ALD are very limited such as use of corticosteroids. However, these therapeutic drugs are not effective for long-term usage. Therefore, additional effective and safe therapies to cope with ALD are urgently needed. Previous studies confirmed that edible food plants and their bioactive compounds exert a protective effect against ALD. In this review article, we summarized the hepatoprotective potential of edible food plants and their bioactive compounds. The underlying mechanism for the prevention of ALD by edible food plants was as follows: anti-oxidation, anti-inflammation, lipid regulation, inhibition of apoptosis, gut microbiota composition modulation, and anti-fibrosis.

## 1. ALD: Epidemiology and Risk Factors

From an epidemiological point of view, intake of alcohol documented as the amount of alcohol consumed in liters per person or per capita and alcohol consumption per capita contributes inaccurate estimation of alcohol use [1]. For instance, alcohol consumption data are mostly obtained from surveys of self-reported intake which may cause underestimation of exact alcohol intake. Additionally, worldwide studies of the disease burden mostly used ICD (International Statistical Classification of Diseases and Related Health Problems) codes, which are uncertainly used in the world. Therefore, the use of ICD codes may lack reports of those who do not seek medical care, consequently failing to report a large proportion of the drinking population. It is believed that around a quarter of the total alcohol consumed worldwide is undocumented [2,3,4].

According to the data of the World Health Organization, the average alcohol intake per person aged 15 years and older was about 6.2 L/year and 13.5 g/day [5]. Peacock et al. [6] reported that in 2015, global estimated prevalence of chronic alcohol intake over the past 30 days was 18.30% in adults. Former Soviet Union and Eastern Europe consume higher amounts (>10 L/capita) of alcohol compared with other regions. Lowest alcohol intake has been documented in the region of Southeast Asia and the Middle East, which was around less than 2.5 L/capita, likely due to significant Islamic populations in these areas. The alcohol type being consumed also differs by geographic region. Worldwide, the most common alcohol intake is wine (8.0%), beer (34.8%), and spirits (50.1%) [5]. Approximately, 67.3% of the United States population (age > 18) consumes alcohol each year, and only 7.4% meet the criteria for alcoholism [7]. Data from the U.S. National Institute on Alcohol Abuse and Alcoholism reported an increase (per capita) in the consumption of alcohol from 2.14 to 2.18 gallon/year in 2001 and also in China from 4.9 to 6.7 L between 2003 and 2010 [5,8,9]. Many factors, such as type of alcohol, age, drinking pattern, gender, ethnicity, obesity, genetics, and smoking, etc., are directly linked with the progression and development of ALD [6,7,8,9].

### 1.1. Alcohol Metabolism

Alcohol is metabolized in the liver in three different ways. It is mainly associated with cytosolic alcohol dehydrogenase (ADH), which catalyzes the oxidative metabolism of alcohol to acetaldehyde. During this pathway, transfer of hydrogen from alcohol toward nicotinamide adenine dinucleotide (NAD) occurs, which is further converted to the reduced form, ultimately producing acetaldehyde. The overproduction of reducing equivalents (NADH) in the cytosol leads to the disturbance of redox potential. Some cytosolic hydrogen equivalents are transferred to mitochondria by many shuttle systems [10,11]. 

The alcohol metabolites of acetaldehyde are further oxidized to acetate via mitochondrial aldehyde dehydrogenase (ALDH2), which occurs in the liver. Alcohol metabolites (acetaldehyde and acetate) are excreted from the liver into the blood and further metabolized peripherally [12]. It has been reported that patients exposed to more alcohol have higher levels of acetaldehyde in their blood compared to non-drinkers [13]. The acetaldehyde metabolism generates a higher amount of NADH in hepatic mitochondria, which further reduces the β-oxidation of long-chain fatty acids by suppressing the activity of long-chain 3-hydroxyacyl-CoA dehydrogenase. Higher alcohol intake leads to elevated levels of acetaldehyde, which is highly toxic in various ways, such as oxidative stress, adduct formation with proteins, glutathione depletion, and lipid peroxidation [14]. Chronic alcohol intake triggers ADH-associated alcohol metabolism to some extent. It has been reported that ADH activity was not elevated, but a hypermetabolic state may be an underlying mechanism [15]. 

**Figure 1:**
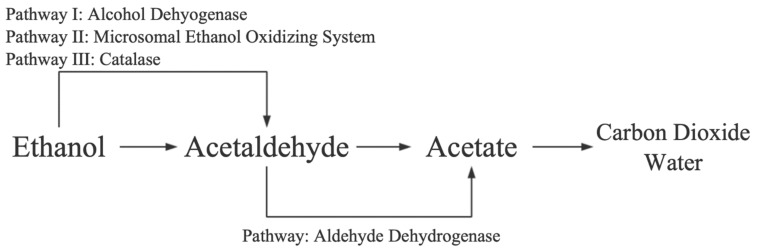
Schematic representation of metabolic pathway of ethanol. Adapted from ref. [18].

The microsomal ethanol-oxidizing system is a cytochrome P-450-dependent pathway, and P-450 2E1 (CYP2E1) is the main substance involved in this pathway. In addition, ethanol may also affect CYP1A1, CYP3A, and CYP4A activities. Chronic alcohol consumption accelerates the activity of CYP2E1, which is the main potential mechanism for improving blood alcohol clearance [12,15,16,17]. Catalase (CAT) is a peroxidase enzyme present in the liver cells and is also involved in alcohol metabolism. Catalase (CAT) metabolizes ethanol into acetaldehyde by conversion of hydrogen peroxide (H_2_O_2_) to 2H_2_O (Figure 1) [13].

Metabolism of alcohol may result in lactic acidosis and decreased renal function for the excretion of uric acid, which further lead to hyperuricemia. It is also reported that alcohol metabolism impaired carbohydrate metabolism with decreased gluconeogenesis from amino acids and caused hypoglycemia. Alcohol metabolites also lead to steatosis, decreased oxidation of fatty acids, increased α-glycerophosphate and triglycerides synthesis, and accumulation of fat in the hepatocytes [14].

### 1.2. Mechanisms of Alcoholic Liver Disease

**Figure 2:**
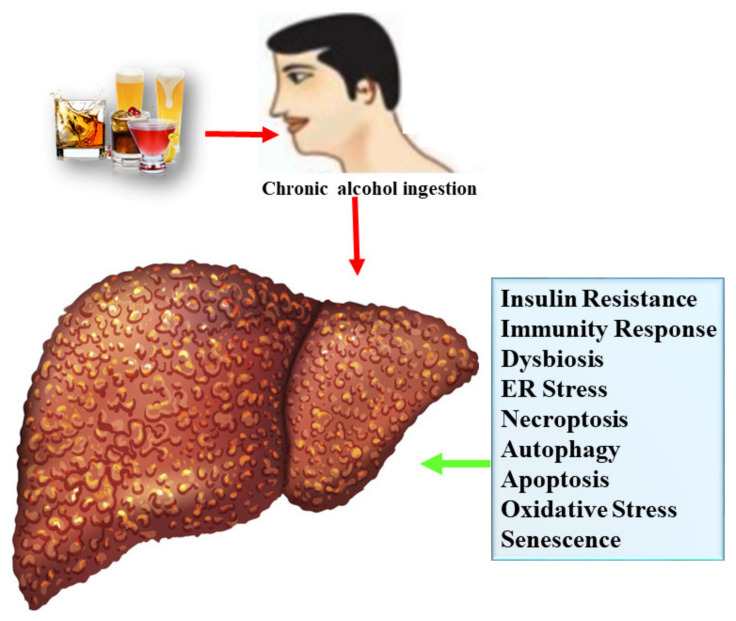
The spectrum and pathogenesis of ALD.

Alcoholic liver disease (AFL) is characterized by a wide range of hepatic disorders, including simple steatosis/AFL disease, complicated lesion of liver damage, steatohepatitis, cirrhosis/fibrosis, and hepatocellular carcinoma [19]. Further details are mentioned below (Figure 2).

Alcohol-induced fatty liver: The excessive consumption of alcohol could promote the storage of fats (triglycerides, cholesterol esters, and phospholipids) in the hepatocytes, leading to AFL disease. It is reported that alcohol and its metabolic product acetaldehyde are not directly involved in the synthesis of fatty acid, while acetaldehyde metabolite acetate is decomposed into acetyl-CoA, which directly assists in the synthesis of fatty acid. Chronic alcohol consumption may facilitate fat accumulation in the liver in multiple ways [12]: (a) alcohol intake increases the ratio of NADH/NAD^+^ in hepatocytes, which disrupts β-oxidation of fatty acids in the mitochondrion and leads to steatosis [20]; (b) alcohol intake increases the hepatic sterol regulatory element-binding protein-1c (SREBP1c) expression, which further triggers lipogenic gene expressions [21]; (c) alcohol intake inactivates peroxisome proliferator-activated receptor α (PPARα), thereby upregulating the expression of many genes involved in oxidation and free fatty acid (FFA) transport [22]; and (d) alcohol intake suppresses AMP-activated protein kinase (AMPK) pathway, which plays a role in inhibiting the synthesis of fatty acid but contributing to fatty acid oxidation [14]. Besides fat metabolism, alcohol intake also affects fatty acid clearance and mobilization. Alcohol intake leads to lipolysis and adipocyte death, leading to an increase in circulating fatty acids and following hepatic accumulation [23]. Alcohol intake also elevates the supply of lipids transported from the small intestine to the liver [20]. If patients do not receive any treatment in the early stages of AFL disease, hepatic fibrosis and cirrhosis may develop and, in severe cases, even result in liver failure [24]. 

Alcoholic steatohepatitis: Alcohol-related steatohepatitis is characterized by hepatic injuries associated with steatosis. Alcohol-related steatohepatitis includes varying degrees of steatosis, bubbling, and lobular inflammation. Other lesions such as alcoholic froth degeneration, acute cholestasis, fibrous occlusion, and inflammatory lesions are also seen in alcoholic steatohepatitis [24,25]. 

Alcohol-induced hepatitis: Alcohol-induced hepatitis (AH) is an acute inflammatory hepatic disease with high morbidity and mortality. AH mostly arises in those patients who have a history of chronic liver disease, where jaundice and complications can develop rapidly. Notably, AH is not linked to alcohol dose. This is why AH occurs only in 10–35% of chronic drinkers. AH is directly related to liver dysfunction and hepatic duct formation. Moreover, previous studies have reported that lipopolysaccharide (LPS) and hepatocyte proliferation have also been shown to be the cause of AH [24,25,26].

Alcohol-induced hepatic cirrhosis or cancer: Hepatocellular carcinoma (HCC) is the third leading cause of cancer-related death in the world. International Agency for Cancer Research reported that alcohol intake, ethanol, and acetaldehyde have carcinogenic effects in humans. Alcohol intake is responsible for oral, bowel, and liver cancer [24,26]. However, the mechanisms associated with alcohol intake and cancer are not clear yet, but many factors can be speculated, such as acetaldehyde, cytochrome P4502E1, malnutrition, localized effects of alcohol, angiogenesis, and alternation in the methylation [24,27,28]. Alcohol plays an important role in triggering cancer by increasing the expression of many oncogenes, followed by cancer-causing mutations [28]. Hepatic stellate cell activation is an important step in the development of hepatic fibrosis. Mesenchymal stem cells, bone marrow cells, and hematopoietic stem cells are used to treat cirrhosis [28,29].

#### 1.2.1. Lipogenesis

The oxidation of ethanol and acetaldehyde results in the production of higher NADH levels, which further alters the redox potential in the cell and triggers lipid synthesis, i.e., lipogenesis [20]. However, the rapid storage of fat in the liver is influenced not only by ethanol-induced redox changes but also by other factors. A study conducted by Osna et al. [30] confirmed that ethanol-induced fat accumulation is multifactorial, for example, increased hepatic lipogenesis stimulated by ethanol, reduced rate of hepatic lipid breakdown by ethanol, and defective hepatic lipid export due to ethanol. In detail, steatosis linked to chronic alcohol consumption is associated with AMPK-related pathways. Chronic alcohol consumption inhibits AMPK signal, which further activates SREBP1c and acetyl-CoA carboxylase (ACC), inhibits PPARα, and finally results in fat accumulation. SREBP-1c has deleterious effects by increasing the biosynthesis of fatty acid via fatty acid desaturation enzymes such as fatty acid synthase (FAS) and stearoyl-CoA desaturase (SCD1) [21]. Contrarily, PPARα is a key transcription factor that alters triacylglycerol accumulation and enhances enzymatic defense against oxidative stress (OS) by activating fatty acid oxidation in alcohol-fed mice. The expression of PPARα is downregulated in hepatocytes due to alcohol intake [31]. Furthermore, AMPK inhibition can activate ACC and increase the amount of malonyl-CoA to promote lipid synthesis, while malonyl-CoA inhibits carnitine palmitoyl transferase 1 (CPT-1), resulting in abnormal fatty acid uptake and mitochondrial β-oxidation inhibition [14].

#### 1.2.2. Oxidative Stress

Alcohol intake causes OS, which is mainly mediated by the generation of reactive oxygen species (ROS). ROS may bind to proteins and result in their functional and structural change, producing neoantigens [32]. Furthermore, ROS can directly bind and impair DNA or result in lipid peroxidation due to the production of malondialdehyde (MDA) and 4-hydroxynonenal (lipid peroxidation product). The lipid peroxidation product then binds to the DNA base and produces etheno-DNA adducts [33,34]. Alcohol-associated ROS generation is stimulated by two pathways: (a) induction of CYP2E1 by alcohol intake and (b) alcohol-mediated inflammation [34,35]. CYP2E1 increases the activity of NADPH oxidase, thus accelerating the mitochondrial transport of reduced form of NADH, which is directly linked with the elevation of electron leakage from the hepatocyte mitochondrial respiratory chain, and ultimately leads to the production of ROS [36]. In alcohol-mediated inflammation, tumor necrosis factor (TNF) production may contribute to the association between N-acetylsphingosine and mitochondria, which in turn leads to ROS generation [37]. Furthermore, production of reactive nitrogen species/nitrosative stress (RNS) can also be elevated after alcohol consumption. In an animal model study (rats), alcohol intake triggered inducible nitric oxide synthase, which further led to the formation of reactive peroxynitrite [38]. The CYP2E1 level was increased during chronic alcohol consumption, and its activity accelerated even after 1 week of chronic alcohol intake (40 g of alcohol/day) [39]. Furthermore, it was also reported that alcohol and iron may act synergistically to generate ROS and OS, which were involved in liver injury. Chronic alcohol consumption accelerated liver iron by elevating absorption from the duodenum conciliate by lowering hepcidin concentration [40].

#### 1.2.3. Inflammatory Response

Chronic inflammation possesses deleterious effects, whereas acute inflammation is involved in homeostasis maintenance in response to noxious stimuli. Alcohol and its metabolites including ROS, acetaldehyde, acetate, and intestinal microbial-derived LPS (lipopolysaccharide) play an important role in the inflammation of ALD [41]. The intestinal microbial-derived LPS induces Toll-like receptor 4 (TLR4) expression, which further activates the nuclear factor-κB (NF-κB), starting the secretion of pro-inflammatory cytokines and mediators, resulting in the necrosis of hepatocytes [42]. Chronic alcohol consumption may also lead to the activation of innate immunity and neutrophil infiltration. The parenchymal neutrophil infiltration in the liver is a typical feature of AH. Parenchymal and nonparenchymal cells of AH patients can trigger the production of chemokines and cytokines in the liver, which ultimately promotes neutrophil infiltration, thus further damaging the liver [43]. It is well known that LPS activates Kupffer cells by TLR4-dependent pathway in the progression of AH [44]. After activation, Kupffer cells release macrophage inflammatory protein-2 and monocyte chemoattractant protein-1 as chemokines and proinflammatory cytokines (IL-6, TNF-α, and IL-1) [41]. Activation of NF-κB is known to be a marker of ethanol-induced hepatotoxicity [45]. It has been reported that cyclooxygenase-2-mediated overproduction of prostaglandin E2 is directly linked with the development of inflammation [46]. It was also reported that induction of proinflammatory cytokine and hepatocyte steatosis is also conciliated by the spleen tyrosine kinase (SYK) activation in inflammatory hepatic hepatocytes and mononuclear cells in mice treated with chronic alcohol. Furthermore, activated SYK kinases were also observed in the liver biopsy samples from ALD patients [47].

#### 1.2.4. Gut Microflora

The gut microbiota is a complex community of microorganisms in the gastrointestinal tract that plays a key role in nutritional digestion and absorption and metabolic and immunological function related to host health and disease [48]. It is reported that alcohol consumption can cause gut microbiota disturbance, elevating the Gram-negative bacteria, damaging intestinal barrier integrity due to endotoxin produced by Gram-negative bacteria, augmenting intestinal mucosa permeability, and reducing bacteria of short-chain fatty acid production [48,49,50]. Endotoxin is part of the cell membrane of Gram-negative bacteria. LPS, an active component of endotoxin, is associated with TLRs and can trigger an inflammatory cascade. Steatohepatitis characteristics were also observed in LPS-treated mice [51,52]. It has been reported that LPS and dead bacteria shed from the cell wall of viable organisms and play a key role in circulating endotoxins. Kupffer cells in the liver detoxify endotoxins via phagocytosis but during the overaccumulation of endotoxins which overwhelms the phagocytotic capacity of Kupffer cells and endotoxins spill into the systemic circulation. Notably, endotoxemia is a condition in which plasma endotoxin level increases to more than 2.5 endotoxin unit/mL. Previously, it was observed that ALD patients exhibit a higher level of plasma endotoxins compared to healthy subjects [53,54,55].

By using various models, the scientists confirmed that chronic alcohol consumption (20%) may be directly associated with the dysbiosis of gut microbial composition. In an animal study, rats fed with 20% of alcohol for 13 weeks showed an increase in *Bacterioidetes* and a decrease in α and β diversity and *Lactobacilli* abundance [56]. In another study, the abundance of phylum *Bacteroidetes* and α-diversity of fecal microbiota decreased in mice fed with short-term ethanol (0.8 g/kg/day for 7 days) [48]. In a human study, the abundance of phylum *Proteobacteria* increased and the abundance of *Faecalibacterium* decreased with a high amount of alcohol intake (118.9 g/day for >10 years) compared to the control group (consumed 2.5 g/day) [50]. Until now, many studies have demonstrated that chronic alcohol consumption directly affects gut microbes, leading to intestinal dysbiosis and inflammation.

#### 1.2.5. Endoplasmic Reticulum Stress

The endoplasmic reticulum (ER) is a cellular organelle that exists in all eukaryote cells and plays an important role in intracellular lipid synthesis, protein synthesis/processing, calcium storage, etc. The depletion in calcium or excessive storage of misfolded/unfolded proteins in the ER lead to ER stress [57]. Chronic alcohol consumption accelerates ER stress by ROS accumulation, increases hepatic CYP2E1 expression and epigenetic, high serum homocysteine level, and reduces the ratio of S-adenosylmethionine to S-adenosylhomocysteine in the liver [58,59]. In ALD, upregulated expression of C/EBP homologous protein (CHOP), caspase-12, and glucose regulatory proteins GRP78 and GRP94 is linked with unfolded protein response (UPR)/ER stress [60]. The activation and upregulation of ER-localized transcription factors such as SREBP-1c and SREBP-2 were directly linked with the increment of lipid accumulation and fatty liver in alcohol exposure [61]. Homocysteine, another important ER stress inducer, was higher in the results of alcoholic human subjects, and hyperhomocysteinemia was also reported in alcohol-fed mice [62].

#### 1.2.6. Apoptosis

Apoptosis is a type of cell death that is highly regulated and controlled by two distinct molecular pathways: (a) internal pathway that relies on mitochondria and (b) exogenous pathway conciliated by death receptors [63]. The death receptors are highly expressed in hepatocytes, and therefore the liver is mainly susceptible to external apoptosis. Prolonged drinking triggers the release of inflammatory cytokines such as FasR and TNF-α. Cytokines binding and death receptors further accelerate the apoptotic process, inducing caspase-8 activation by the caspase cascade. After that, caspase-8 stimulates caspase-3, -6, and -7, ultimately leading to apoptotic cell death. ROS and CYP2E1 are another mechanism of ethanol-induced hepatocyte apoptosis. ROS mainly activates pro-apoptotic members of the Bcl-2 (B-cell lymphoma-2) family through intrinsic pathways and oligomerizes on the mitochondrial outer membrane, leading to mitochondrial dysfunction and activation. The starter caspase-9 is further activated by P450 and activates caspase-3, -6, and -7 after mitochondrial secretion, which are responsible for cellular substrate degradation [64]. Furthermore, various experimental studies (in vivo and in vitro) have reported that higher alcohol exposure increases cell death by apoptosis [65,66].

## 2. Bioactive Compounds Ameliorate ALD via Multiple Pathways

### 2.1. Flavonoids

#### 2.1.1. Flavonols

Flavoniods including flavonols has been reported to possess multiple health benefits such as protect liver from ALD, NAFLD, intestinal inflammations and others [67,68,69,70]. Quercetin (3,3′,4′,5,7-pentahydroxyflavone) is one of the most common dietary flavonols present in the apple, onion, black or green tea, herbal medicine, and red wine [69,70]. Many studies have reported that quercetin exerts a beneficial role in non-alcoholic fatty liver disease (NAFLD), ALD, and diabetes mechanically by regulating the expression of lipid-metabolism-related genes and the secretion of inflammatory mediators [69,70,71,72,73]. Quercetin blocked autophagy suppression induced by ethanol. Moreover, it also promoted lipophagy by increasing the co-localization of hepatic microtubule-associated protein 1 light chain 3II (LC3II) and perilipin 2 (PLIN2) proteins, activating AMPK activity, and decreasing PLIN2 level [69]. In another study, quercetin attenuated ALD in a similar way as reported by Zeng et al. [69]. Briefly, quercetin decreased the accumulation of LC3II and p62, increased LAMP (1 and 2) and Rab7, and regulated the mTOR-TFEB pathway, which was the major underlying mechanism by which quercetin ameliorated lysosomal autophagy dysfunction induced by ethanol [74]. In an in vitro study, quercetin, quercetin-3-glucoside, and rutin significantly improved ethanol-induced liver injury via NF-E2-related factor 2 (Nrf2)/antioxidant response element (ARE) pathway [70].

The protective effect of fisetin on ALD was mainly due to effects of improving OS and accelerating ethanol scavenging [75]. Similarly, in another study, fisetin reversed alcohol-induced liver injury by increasing antioxidant defense and regulating mitochondrial respiratory enzymes and matrix metalloproteinase activities (MMPs) [76]. Many studies have shown that MMPs are involved in the pathogenesis of ALD [77]. Specifically, MMPs were involved in liver regeneration and remodeling of extracellular matrix primarily via the degradation of their components such as collagen, gelatin, and fibronectin. Furthermore, MMP-9 and MMP-2 expression were found to be elevated in liver fibrosis/cirrhosis [78]. Dihydromyricetin can alleviate liver injury induced by alcohol by suppressing inflammation responses, changing lipid metabolism, and increasing ethanol metabolism [79]. A study demonstrated that morin can reverse alcohol-induced liver injury by improving OS and decreasing hepatic function enzymes [80].

Kaempferol belongs to flavonol group compounds and is widely distributed in fruits, teas, vegetables, and medicinal herbs. It was reported that kaempferol enhanced intestinal barrier function by increasing the tight junction proteins in Caco-2 cells. In addition, kaempferol directly protected liver tissues from alcoholic liver injury in animal models [81]. In another study (in vitro and in vivo), we noticed that kaempferol significantly reduced the expression and activity of hepatic CYP2E1, which was identified as a key microsomal enzyme involved in ALD [82,83]. More interestingly, a recent study also confirmed that kaempferol supplementation reversed the deleterious effect of alcohol and may serve as a prophylactic treatment toward ALD by elevating the expression of butyrate receptors, tight junction proteins, and transporters in the intestinal mucosa of experimental mice [84].

#### 2.1.2. Isoflavones

Isoflavones are abundantly present in soybeans and soy products and have been reported to protect the liver from alcohol-associated injuries. Genistein and puerarin were also studied against chronic alcohol-induced liver injury. The results reveal that both isoflavones (genistein and puerarin) significantly balance alanine aminotransferase (ALT), hepatic antioxidant capacities, apoptosis, inflammation, and serum and hepatic lipids in ethanol-administered (50%, *v*/*v*) mice [85]. The results of Leelananthakul et al. [86] were well matched with Zhao et al. [85]. They observed that genistein alleviated alcohol-induced liver injury in rats via antioxidant and anti-inflammatory mechanisms [86]. In addition, tectoridin, an isoflavone glycoside, also led to a certain improvement in ethanol-induced liver steatosis and injury [87,88].

#### 2.1.3. Flavones

Luteolin (3′,4′,5,7-tetrahydroxyflavone) was studied for the possible treatment of ALD. The results show that ethanol increased the expression of lipogenic genes such as *Srebp1c*, *Fasn*, *Acc*, and *Scd1*, which were downregulated by luteolin. Furthermore, ethanol reduced AMPK activation and SREBP-1c phosphorylation, which were abrogated by luteolin. It was concluded that luteolin was effective in alleviating alcohol-induced liver injury and steatosis [89]. Wogonin is a flavonoid present in many food plants and shows anti-tumor and anti-inflammatory activities. Wogonin elevated the expression of peroxisome proliferator-activated receptor γ (PPARγ) and decreased the activity of NF-κB p65 to alleviate the inflammatory response of ALD [90].

Baicalin, a flavone compound, has been demonstrated to have hepatoprotective effects by modulating OS, inflammation, and fibrosis [91]. It was reported that baicalin can alter the expression of Nrf2 and NF-κB transcription factors, as well as regulate CYP2E1 activity, which were key regulators of antioxidant and inflammatory defense in ALD [92]. Simultaneously, evidence indicated that baicalin can ameliorate ALD by regulating the Sonic hedgehog pathway (an important pathway involved in liver injury and tissue repair) [93].

Diosmin is a kind of flavone that exists abundantly in citrus fruits. Evidence documented that diosmin can attenuate alcohol-induced liver injury mainly via regulating the activation of TNF-α and NF-κB. In addition, another study showed that apigenin also significantly attenuated ALD by regulating hepatic CYP2E1-mediated OS and PPARα-associated lipogenic gene expression [94]. Chrysin, a natural bioactive compound abundantly present in honey, propolis, and many plants, can protect against ethanol-mediated oxidative stress [95].

#### 2.1.4. Flavanones

Naringin is a flavanone-7-O-glycoside abundantly present in citrus fruits (grapefruit) that imparts the bitter taste to fruits and possesses many health benefits. Zhou et al. [96] conducted a study to determine the beneficial effect of naringin in the treatment of alcoholic liver injury. The results show that naringin could markedly inhibit alcoholic liver steatosis and damage by reducing OS, apoptosis, and lipid accumulation. Similarly, in another study, naringenin attenuated alcohol-induced liver steatosis and injury by reducing DNA damage and apoptosis in zebrafish larvae [97]. It was documented that hesperidin could protect from the deleterious effect of alcohol by regulating alcohol and lipid metabolism, reducing DNA damage and ER stress [98].

#### 2.1.5. Flavan-3-ols

Previous evidence documented that flavan-3-ols (catechins and epigallocatechin-3-gallate, etc.) could protect the liver from the harmful effects of alcohol. In detail, a study was conducted to explore the protective effect of epigallocatechin-3-gallate (EGCG) against alcohol-induced liver injury in experimental rats. The rats were administered with alcohol and EGCG for 6 weeks, and various biochemical indicators were determined. It was observed that EGCG administration blocked the gut leakiness and decreased endotoxemia, lipid peroxidation, and liver inflammatory indicator [99]. Yun et al. [100] also confirmed that EGCG attenuated alcohol-induced hepatotoxicity and fatty liver. This effect was due to improvements in CPT-1 and phosphorylated ACC levels, which inhibited the development of fatty liver. In another study, EGCG was reported to alleviate the detrimental effects of ethanol [101]. Rishi et al. [102] concluded that EGCG and *L. plantarum* synergistically protected against alcohol-associated toxicity. Bharrhan et al. [103] studied the effect of catechin on ethanol-induced liver injury in rats. The results conclude that catechin can ameliorate ethanol-induced liver injury by suppressing the induction of NF-κB (the key component of the signaling pathway and associated with liver inflammation). A recently published study also confirmed the protective effect of EGCG against alcohol. It hypothesized that this surprising effect of EGCG may be due to the direct interaction of EGCG with hepatic Kupffer cell TLR2/3 receptors and modulating IL-10 signaling, thus exerting divergent effects on ethanol-induced liver damage [104].

#### 2.1.6. Anthocyanin and Proanthocyanin

Anthocyanins are purple, blue, or red pigments abundantly present in plants (especially fruits, flowers, and tubers). Anthocyanins belong to the polyphenolic compound group flavonoids. Previously, it was reported that black rice anthocyanin (peonidin-3-glucoside, cyanidin-3-glucoside, cyanidin-3,5-diglucoside, and cyanidin-3-rutinoside) extract can markedly improve the adverse effect of alcohol in experimental rats [105].

Cyanidin-3-glucoside (C-3-G) is the most abundant monomer of anthocyanins, which is commonly present in fruits and vegetables. Wan et al. [106] reported the beneficial effect of C-3-G against ALD. C-3-G administration (200 mg/kg bw for 12 weeks) reversed the liver injury in mice fed a high-fat and high-cholesterol diet plus ethanol. A recently published study also confirmed that C-3-G shows strong potential to overcome the adverse effect of alcohol. It found that C-3-G alleviated alcoholic steatohepatitis by inactivating NLRP3 inflammasome and Sirtuin1 (SIRT1)/NF-κB signaling pathway [107].

Liver fibrosis is an advanced stage of liver injury caused by alcohol. It was reported that a high-fat diet with ethanol in drinking water increased the expression of collagen α, α-smooth muscle actin, transforming growth factor-β (TGF-β), and tissue inhibitors of metalloproteinases (TIMP) 1 and 2, induced lipid accumulation, and increased OS, autophagy, and inactivation of AMPK pathway, whereas anthocyanin supplementation rebalanced these parameters. The protective effect of anthocyanins against ALD and alcoholic liver fibrosis was mainly due to the regulation of AMPK/mTOR/autophagy pathway [108]. Blueberry was also reported to significantly decrease liver weight and hepatic lipids in ethanol-induced liver damage [109]. Zhu et al. [110] also reported a similar result. Anthocyanins-enriched blueberry juice combined with probiotics markedly suppressed the apoptosis in ALD mice by affecting SIRT1 signaling pathway.

Blue honeysuckle (*Lonicera caerulea* L.) has multiple pharmacological effects and is widely distributed in North Korea, China, and Japan. Blue honeysuckle consists of a high content of anthocyanins (C-3-G and peonidin-3-glucoside). Blue honeysuckle was also studied for the possible treatment of ALD. The results show that administration of blue honeysuckle at the dosage of 80 and 150 mg/kg bw could significantly reduce the adverse effect of ethanol in mice. In terms of mechanism, their results also report that blue honeysuckle extract alleviated alcoholic hepatosteatosis by activating AMPK signaling pathway [111]. Anthocyanins-enriched black chokeberry (*Aronia melanocarpa*) extracts could also prevent ALD by suppressing OS and Nrf2 signaling pathway [112].

Proanthocyanins were also reported to exhibit protective effects against ALD. Previously, oligomeric proanthocyanins were administered to female C57BL/6 mice for 9 days along with alcohol, and the results show that oligomeric proanthocyanins attenuated liver steatosis and injury by decreasing the activities or levels of ALT, aspartate aminotransferase (AST), triglycerides (TG), total cholesterol (TC), low-density lipoprotein cholesterol, and MDA and increasing the activities or levels of high-density lipoprotein cholesterol and superoxide dismutase (SOD). In addition, oligomeric proanthocyanins markedly downregulated the expression of inflammatory cytokines (TNF-a, IL-1β, and IL-6) and lipid synthesis genes (SREBP-1c and SREBP-2). Moreover, the underlying mechanism regarding alleviating liver steatosis and damage by oligomeric proanthocyanins was also determined on an AML-12 cells line. The results show that oligomeric proanthocyanins could activate AMPK and remarkably improve liver injury induced by ethanol [113].

### 2.2. Alkaloids

Alkaloids such as berberine and nuciferine were demonstrated to have a protective effect against ALD. In detail, an in vivo study conducted by Zhang et al. [114] confirmed the protective effect of berberine (BBR) against ethanol-induced liver injury. Treatment of BBR (200 and 300 mg/kg bw for 10 days) significantly attenuated OS, mitochondrial oxidative damage, and macro steatosis and blunted the lipid accumulation in ethanol-induced mice. These results conclude that BBR could be an ideal candidate for the treatment of ALD [114]. A recently published article reported that BBR ameliorated ALD by modulating several pathways. Briefly, BBR significantly enhanced the increase of granulocytic-myeloid-derived suppressor cells (G-MDSCs)-like cells in the liver and blood and downregulated T cells. Repression of the G-MDSCs-like population markedly ameliorated the protective effect of BBR against alcohol. BBR activated IL-6/signal transducer and activator of transcription 3 (STAT3) signaling pathway, whereas suppression of STAT3 activity improved the G-MSDCs-like population activation by BBR. Additionally, it was also reported that BBR changes the overall gut microbial composition, mainly by increasing the abundance of *Akkermansia muciniphila*. Collectively, their results reveal that BBR attenuates ALD by modulating the gut microbiota and immunosuppressive response in ALD [115].

In another study, alkaloid nuciferine isolated from *Nelumbo nucifera Gaertn* (also named lotus) was tested against ALD. The results reveal that nuciferine could activate Nrf2/heme oxygenase 1 (HO-1) signaling in HepG2 hepatocytes and protect against alcohol-induced ROS generation. Of note, nuciferine ameliorated alcohol-induced liver injury by activating miR-144/Nrf2/HO-1 pathway, alleviating OS, and reducing inflammation [116].

### 2.3. Chalcone and Anthraquinone

Chalcones are a class of polyphenols commonly found in edible plants, fruits, and vegetables. Chalcones are precursors of isoflavonoids and flavonoids with a wide array of biological activities including anti-ALD. Hydroxysafflor yellow A (HSYA) was screened against ALD. The results conclude that HSYA could effectively protect the liver from long-term alcohol injury in rats by increasing hepatic antioxidant capacity and inhibiting the expression of TGF-β1 [117]. Similarly, chalcone derivative L6H21 could reduce ethanol-induced hepatic inflammation in mice [118]. Anthraquinone (emodin and chrysophanol) showed hepatoprotection in HepG2 induced by ethanol. In another study, Liu et al. [119] also observed the hepatoprotective effect of emodin on ethanol-induced fatty liver injury in mice.

### 2.4. Lignans

Lignans are bioactive compounds produced by shikimic acid biosynthetic pathway in many plant species. Lignans are commonly present in plant seeds, leaves, roots, fruits, rhizomes, flowers, stems, and resins. Flavonolignan (Silymarin) was also reported to possess a hepatoprotective effect against ALD [120,121]. In a large randomized controlled trial before liver transplantation and before the discovery of the hepatitis C virus, long-term treatment with silymarin could decrease the mortality of patients with cirrhosis, especially those exposed to ethanol [122]. In another small randomized controlled trial, silymarin exhibited a slight decrease in lipid peroxidation and an increase in glutathione in peripheral blood cells in patients with alcoholic liver cirrhosis [123].

*Schisandra chinensis* (Turcz.) Baill. (SC) is a traditional Chinese plant that is commonly used for nutritional and medicinal purposes. Lignans derived from SC or lignans-enriched SC extracts exhibited beneficial effects on the liver. For example, Wang et al. [124] observed that lignans-enriched SC extracts (Schizandrol A and Schizandrol B) significantly ameliorated alcohol-induced liver damage by improving serum and liver biomarkers in male ICR mice exposed to 50% (*v*/*v*) alcohol for 60 days. Similarly, a recent study conducted by Su et al. [125] also found a surprising effect of SC against ALD. Daily administration of SC (400 and 800 mg/kg bw) for 14 days significantly ameliorated alcohol-induced liver damage in rats by suppressing CYP2E1 activation and activating the Nrf2/antioxidant response element signaling pathway. In another recently published study, lignans-enriched SC extracts were shown to ameliorate alcohol-induced long-term liver injury and decrease hepatocellular degeneration by blocking endothelin receptor B [126].

### 2.5. Saponins and Terpenoids

Saponins and terpenoids are the largest group of bioactive compounds, with a total of more than 40,000 compounds. Previous evidence reported that saponins and terpenoids from many plant species are effective in the treatment of ALD. Maslinic acid was a kind of pentacyclic triterpene that was reported to attenuate ALD. Briefly, maslinic acid rebalanced the activities or expression of ALT, AST, CYP2E1, C reactive protein, and antioxidant indicators in an ALD rat model. In addition, maslinic acid was also reported to markedly improve alcohol-induced liver injury via OS and inflammatory pathways [127]. Similarly, pentacyclic triterpenoid carboxylic acid (ursolic acid) was also reported to improve the morphological changes mediated by alcohol and normalized liver microanatomical changes [128].

Dioscin, a kind of steroidal saponin, was studied against ALD. The results show that dioscin could ameliorate alcohol-induced liver disease by ameliorating the production of inflammatory cytokine, OS, and hepatic steatosis, which may contribute to the development of drugs against alcoholic liver injury [129,130].

The roots and leaves of *Panax ginseng* C.A. Mey. and *Panax notoginseng* (Araliaceae), well-known Chinese herbal plants, have been extensively used for the treatment of various diseases. Previous studies have confirmed that saponins-enriched leaves and flower buds of *Panax notoginseng* could protect against acute and chronic ethanol exposure. These pharmacological activities of *Panax notoginseng* were due to the abundant presence of ginsenosides Rc, Rb2, and Rb3 [131,132,133]. Ginsenoside Rg1 alleviated liver impairment by downregulating serum parameters such as ALT, AST, TG, and lactate dehydrogenase (LDH) in alcohol-induced mice [134]. Oleanolic acid is a kind of triterpenoid that can elevate antioxidant enzyme activities and Nrf2 levels to improve ALD [135]. Gentiopicroside belongs to iridoids (monoterpenoid group), which is a safe and effective choice for the treatment of ALD. Gentiopicroside could reduce the activities or levels of serum transaminase and TG, regulate the expression of PPARα, SREBP-1, phosphorylated ACC in mice, and ultimately prevent liver injury caused by ethanol exposure [136].

### 2.6. Stilbenes

Stilbenes are polyphenolic compounds that are abundantly present in food plants and are a promising candidate for the treatment of NAFLD and ALD [137]. Resveratrol is a stilbene compound present in many grape species and possesses hepatoprotective activities. Evidence shows that resveratrol could significantly attenuate alcohol-induced liver damage mainly by restoring hepatic antioxidant enzymes, apoptosis, and inflammation [138]. A similar result was reported by Lai et al. [139]. Of note, other stilbenes such as cajaninstilbene acid and vaticanol A, etc., are also consistently used in various health-promoting activities. However, data regarding ameliorative effect of other stilbenes against ALD are not present. Future studies should be carried out on isolation and characterization of stilbenes from food plants and their protective effect on ALD.

### 2.7. Tannins

Tannins are water-soluble polyphenols abundantly present in various food plants such as walnuts, raspberries, and grapes, etc., possessing a broad spectrum of health-promoting activities including ALD. Ellagic acid (EA) derived from raspberry was studied against alcohol-induced toxicity in HepG2 cells. HepG2 cells were treated with EA at 1, 10, and 100 µM concentrations, and the protective effect of EA was determined by regulating nitric oxide (NO), SR-B1, and TGF-β1 production. The production of NO, SR-B1, and TGF-β1 in HepG2 cells increased after the treatment of ethanol, and EA markedly restored NO release and TGF-β1 expression but had no effect on the SR-B1 protein. The above results indicate that an EA-enriched extract of raspberry could be beneficial against alcohol-induced hepatotoxicity [140]. Two ellagitannins—geraniin and amariin—were reported to protect the liver from alcoholic toxicity by inhibiting oxidation of lipids and proteins, modulating Bcl-2-associated X (Bax)/Bcl2 ratio, and restoring antioxidant enzymes in mice [141]. Previously, Devipriya et al. [142] reported that EA at a dosage of 60 mg/kg bw exhibited a potent hepatoprotective effect in alcohol-induced experimental animals by maintaining pro-oxidant and antioxidant imbalance. Alcoholic fibrosis and its associated cirrhosis occurred following the increased matrix synthesis and decreased matrix degradation. Hepatic fibroproliferation is directly linked with changes in hepatic tissue inhibitors of matrix metalloproteinases (MMPs) and matrix metalloproteinases (TIMPs). The hepatic matrixin, MMP, and TIMP expressions are key diagnostic markers of end-stage liver disease. The study reported that co-administration of alcohol (20%, *v*/*v*) and EA (30, 60, and 90 mg/kg bw) downregulated the fibrotic markers such as MMP-2, MMP-9, and TIMP-2 compared with alcohol-induced rats [143] (Table 1).

## 3. Multicomponent Food Plant Extracts against ALD

### 3.1. Fruits

Many in vitro and in vivo studies confirmed that fruits such as the mango [144], grape [145], blueberry [110], mulberry [146], persimmon [147], pomegranate [148], cranberry [149], and wolfberry [150] play an important role against ALD. This ameliorative effect on ALD was mainly due to the presence of a tangible amount of bioactive compounds in fruits. Mango (*Mangifera indica Linn.*), also known as the ‘king of fruits’, contains many nutrients and phytochemicals that can mitigate a variety of chronic diseases. The research conducted by Li et al. [144] demonstrated that mangiferin (a bioactive compound present in mango) could alleviate alcoholic hepatitis. The mangiferin markedly regulated the abnormal liver function, FFA, the metabolism of alcohol, and metal elements in serum. In addition, mangiferin can improve the expression of PPARγ, NLRP3, NF-κB p65, and IL-1β in rats. Furthermore, mangiferin treatment downregulated fumarate levels, D-glucurone-6, 3-lactone, cAMP, and xanthurenic acid and upregulated the phenylacetylglycine and hippuric acid, thereby adjusting phenylalanine metabolism, aldarate, ascorbate, and tricarboxylic acid cycle metabolic pathways. These results suggest that mango-derived mangiferin could attenuate alcoholic hepatitis by modulating specific alcoholic-hepatitis-associated genes, biomarkers, and metabolic pathways. The dysfunction in adipose was also closely related to ALD. The impact of mangiferin on ethanol-induced liver injury and adipose dysfunction was studied. The results demonstrate that mangiferin can protect against ethanol-induced adipose hyperlipolysis by restoring PDE3B stability, which is closely linked with AMPK/TBK1 signaling activation, and inhibiting noncanonical NF-κB activation, resulting in FFA release, and finally ameliorating ALD [151].

Pari and Suresh [152] reported that grape (*Vitis vinifera L.*) leaf extract reduced the risk of ALD in an animal experiment. The results further show that grape leaf extract at a high dose of 100 mg/kg bw could significantly reduce lipid peroxidation level along with restoration of enzymic and non-enzymatic antioxidants level in the kidney and liver of alcohol-exposed rats. Recently, Amen et al. [145] also confirmed that polyphenol-rich grape leaf extracts interfered with NF-κB signaling and exerted antioxidant effects, which further played a role in ameliorating apoptosis and associated hepatic injury in rats exposed to ethanol.

Lychee (*Litchi chinensis* Sonn.) is a popular fruit because of its unique color, delicious flavor, and high nutritive value. The whole fruit of lychee is not only used as a food source but also considered for medicinal purposes. In vitro and in vivo studies confirmed that lychee fruits exhibited hypoglycemic, hypolipidemic, anticancer, hypotensive, antioxidant, anti-obesity, anti-atherosclerosis, neuroprotective, hepatoprotective, and immunomodulatory activities. These therapeutical potentials have been attributed to their nutritional components such as polysaccharides and polyphenols [153,154,155]. A research group from China claimed that lychee pulp phenolic extract (LPPE) exhibited protective effects against ethanol-induced liver injury [153]. In detail, LPPE could reduce hepatic steatosis, TG levels, Nrf2 expression, suppress the expression of lipid synthesis genes, elevate the fatty acid β-oxidation genes, and improve the antioxidant status. Furthermore, LPPE markedly decreased serum endotoxin level and balanced intestinal microbial composition. In another study, the same researchers [154] found that LPPE supplement increased mitochondrial membrane potential, mitochondrial DNA content, hepatic ATP level, and activities of mitochondrial complexes (I and IV) and also suppressed mitochondrial 8-hydroxy-2-deoxyguanosine level. Moreover, repression of Bax expression and Bax/Bcl-2 ratio, inhibition of caspase-3 activity and cytoplasmic cytochrome c level, and increased Bcl-2 expression in the liver were also observed in LPPE-treated mice. They concluded that LPPE showed beneficial effects against ALD by alleviating mitochondrial dysfunction [154]. It was also documented that LPPE could reverse alcohol-induced liver injury by improving intestinal barrier dysfunction, intestinal microbiota dysbiosis, and liver inflammation, suggesting that lychee pulp may be an effective strategy to cope with ALD [155].

Berries (blackberry, cranberry, strawberry, mulberry, raspberry, etc.) are the best dietary sources of biologically active compounds, such as flavonoids (flavonols, anthocyanins, etc.), ascorbic acid, phenolic acid, and tannins. All of these components have cumulative or synergistic effects on the treatment of a variety of diseases, including ALD. Blueberry was studied for the possible treatment of alcoholic fatty liver. Blueberry juice (1.5 mL/100 g) combined with a probiotic mixture of *Streptococcus thermophilus*, *Bifidobacterium*, and *Lactobacillus bulgaricus* (20 mL/100 g probiotics) was orally given to alcohol-induced C57/6J mice for 10 days. The results show that the combination of blueberry juice and probiotics significantly improved antioxidant status, suppressed Forkhead Box O1 (FOXO1), acetylated FOXO1, phosphorylated FOXO1, Bcl-1, FasL, Bax, and caspase-3 by increasing the SIRT1 pathway, thus reducing apoptosis in mice with ALD [110]. Another recent study of Zhuge et al. [156] reported that blueberry could promote autophagy, which triggered lipid metabolism, thereby decreasing hepatic steatosis. The protective effect of the blueberry may be due to the abundance of flavonoid compounds.

*Morus*, also known as mulberry, are consumed as raw or processed in marmalades, wine, juices, jams, and vinegars and are also used as traditional herbal medicines. A previous study confirmed that mulberry possessed hepatoprotective effects in both animals and humans [157]. It was also reported that mulberry fruits and leaves could mitigate ALD via multiple pathways such as reducing lipid accumulation and lipid synthesis, decreasing OS, increasing fatty acid transport and fatty acid oxidation responses, and exerting an anti-inflammatory effect [146,158]. Gao et al. [150] confirmed that wolfberry-derived zeaxanthin dipalmitate can protect the liver from alcohol-induced toxicity and concluded that daily intake of wolfberry may have a positive impact on ALD. Simultaneously, various studies also documented that berry fruits such as cranberry [149], blackcurrant berry [159], Indian gooseberry [160], and ginseng berry [161] can attenuate ALD.

Many other fruits such as apricot [162], guava [163], pomegranate [148], lemon [164], *Citrus depressa* [165], noni fruit [166], *Opuntia ficus indica* [167], jujube [168], and persimmon [147] were also reported to mitigate ALD. These findings confirm that daily intake of fruits may be an effective strategy for alleviating ALD.

### 3.2. Vegetables

Vegetables are good sources of various phytochemicals such as polyphenols, carotenoids, ascorbic acid, etc., and play key roles in the management of chronic diseases. Purple sweet potato (PSP) has attracted widespread attention due to its high nutritional value, special color, and ameliorative potential against various diseases. PSP was studied for the possible treatment of ALD. Anthocyanin-enriched (C-3-G and peonidin) PSP extract was intragastrically administered at the dosage of 50, 125, and 375 mg/kg bw to mice combined with alcohol for 30 days. The results show that alcohol led to abnormalities in various biomarkers such as ALT, AST, TC, LDH, MDA, and SOD, whereas the PSP-treated group rebalanced these biomarkers. In addition, histopathological analysis revealed that the liver cell swelling was significantly alleviated, and pathological features were improved in PSP-treated mice [169]. In another study conducted by Jiang et al. [170], PSP improved antioxidant defense by inhibiting CYP2E1 expression, thereby alleviating alcohol-induced liver injury. Results from these two studies confirm that PSP has the ability to protect from the adverse effect of alcohol, which was needed for further research.

Onion (*Allium cepa*) is the most valuable vegetable crop utilized and grown with important pharmacological properties. Onion bulbs are rich in many dietary compounds and bioactive phytonutrients. Previous studies have also reported that onion was effective in the treatment of ALD. Wine made from onion ameliorated ALD in rats [171]. In addition, onion is a good source of sulfur compounds and quercetin. These bioactive compounds are very effective against ALD.

Emerging evidence has reported that garlic and its products such as garlic oil, aged garlic extract, and organosulfur compounds possess therapeutic effects on ethanol-induced liver injury. The garlic products could attenuate ethanol-induced liver injury by improving OS, inhibiting CYP2E1 activity in hepatocytes, regulating lipid metabolism, and improving the gut–liver axis and the adipose–liver axis [172,173,174,175,176,177].

Asparagus (*Asparagus officinalis*) is a widely consumed vegetable with various biological activities. Results show that leaves of asparagus can protect hepatocytes from alcohol-induced toxicity via alleviation of alcohol hangover. Thus, leaves that are mostly discarded possess potential against ALD [178].

Recently, Zhang et al. [179] conducted a study to determine the ameliorative effect of okra seed oil on ethanol-induced liver injury in mice. The okra seed oil was orally administered (400 and 800 mg/kg bw) for 8 weeks to mice combined with alcohol. Results show that the okra seed oil supplementation could inhibit hepatic fat accumulation, reduce inflammatory biomarkers, and improve antioxidant level in ethanol-induced mice. Additionally, okra seed oil also maintained intestinal eubiosis by enhancing the *Bacteroidetes* and reducing *Proteobacteria*, *Clostridium XlVa*, and *Staphylococcus* population in ethanol-induced mice.

Similarly, other vegetables such as artichoke, bitter gourd, and rhubarb could also protect the liver from the toxic effect of alcohol mainly by improving OS, reducing inflammation, and balancing gut microbiota composition [180,181,182].

### 3.3. Spices

Spices are pleasantly aromatic, dried parts of plants such as cinnamon, cloves, saffron, turmeric, ginger, cumin, chili, and black pepper. Spices can protect against a variety of acute and chronic diseases such as ALD. Cinnamon bark extract was administered to mice for four days prior to ethanol [183], and results state that cinnamon bark extract markedly decreased the hepatic lipid storage by inhibiting the expression of the MyD88 and NO. Furthermore, it was also observed that cinnamon bark extract could reduce MyD88 and TNF-α levels in LPS-induced RAW 264.7 cell line [183].

Fenugreek (*Trigonella foenum graecum*) is a kind of spice commonly used in India and other regions. Fenugreek was reported to protect the liver from alcohol-induced damage. Polyphenolic-compound-enriched seed extract of fenugreek attenuated alcoholic toxicity mainly by modulating lipid profile and collagen contents in rat liver [184]. *Crocus sativus* L., known as saffron, is commonly used as a spice in various regions such as Spain, Morocco, and Iran. Recently, Azizi et al. [185] reported that bioactive-compound-enriched *Crocus sativus* L. petal (safranal, crocin, myricetin, and quercetin) could ameliorate ethanol-induced liver damage by reducing inflammatory biomarker.

Similarly, other spices plants such as *Petroselinum crispum* (parsley oil) [186], clove (*Syzygium aromaticum* L.) [187], *Thymus vulgaris* [188], and peppers [189] were also documented to possess a beneficial effect against alcohol-induced liver injury.

### 3.4. Cereals and Grains

The hepatoprotective effects of rice bran polyphenolic-enriched extract were investigated by animal experiments. Mice were fed with rice bran extract along with ethanol for 8 weeks. In the study of Xiao et al. [190], it was reported that rice bran polyphenolic-compounds-enriched extract could mitigate ethanol-induced liver damage by reducing hepatic function markers and lipid profile levels. Additionally, ethanol exposure may result in intestinal microbiota dysbiosis which was further improved by rice bran polyphenolic-compounds-enriched extract. What is more, rice bran polyphenolic-compounds-enriched extract further improved the expression of Reg3g, claudin-1, zonula occludens 1, and claudin-4 induced by alcohol, indicating that rice bran may have an ameliorative effect on intestinal barrier dysfunction. Treatment of rice bran also repressed the alcohol-induced activation of hepatic endotoxin-TLR4-NF-κB pathway and ultimately mitigated liver inflammation. In conclusion, rice bran supplementation can attenuate intestinal microbiota dysbiosis, repress inflammatory responses in the liver, and inactivate the endotoxin-TLR4-NF-κB pathway, which may be an effective way to mitigate ALD [190]. Another recent study conducted by the same research group [191] documented that rice bran polyphenolic-enriched extract showed protective effects against ALD by alleviating mitochondrial dysfunction and led to hepatocyte apoptosis via peroxisome proliferator-activated receptor-gamma coactivator (PGC-1α)-mitochondrial transcription factor A (TFAM) signal pathway mediated by microRNA-494-3p. Earlier, black rice, a good source of anthocyanins, was reported to exert a protective effect against alcohol-induced liver damage. In detail, black rice extract enriched with anthocyanins (cyanidin-3,5-digluco-side, cyanidin-3-glucoside, cyanidin-3-rutinoside, and peonidin-3-glucoside) in rats attenuated liver injury by balancing several biomarkers and improving liver histological features and OS [105].

Buckwheat has two types that are used as food: common buckwheat (*Fagopyrum esculentum*) and tartary buckwheat (*Fagopyrum tataricum*). Recently, a study revealed the hepatoprotective effect of tartary buckwheat on alcohol-induced acute and chronic liver injuries. Their results conclude that tartary buckwheat extract alleviated alcoholic toxicity mainly via OS and mitochondrial cell death pathways [192].

Corn is an important cereal consumed worldwide with tremendous health benefits and is also reported to protect against ALD. In detail, peptide isolated from corn decreased hepatic MDA and TG levels, increased hepatic activity of glutathione, and improved hepatic histological features [193]. A clinical study has determined the beneficial effects of corn in ALD subjects. In that study, 161 alcoholic patients were enrolled and received corn (4 g/day) for 9 weeks. Results show that corn supplementation markedly decreased the serum TC, TG, ALT, AST, MDA, and TNF-α and increased activities of SOD and glutathione peroxidase compared to placebo. In conclusion, corn may have protective effects on ALD by improving OS and modulating lipid metabolism, which can be used as a functional food for the management of alcohol-related disorders [194].

Barley sprouts are young leaves harvested from barley seeds after about 10 days of sowing and have become increasingly popular as functional foods in recent years. It was also documented that barley sprouts could attenuate ethanol-induced liver damage by reducing inflammatory response in RAW 264.7 cells [195]. In addition, GanMeijian, a famous barley product in China, was also reported to ameliorate oxidative damage and lipid accumulation in ethanol-induced Wistar rats. The underlying mechanisms were mainly reducing the accumulation of ROS level in the liver, balancing OS state, protecting hepatic function, elevating mitochondria activity in hepatocytes, and inhibiting the expression of fat synthesis genes [196].

Liu et al. [197] investigated the effects of mung beans on alcohol-induced liver injury in mice, and results show that intake of active constituents isolated from mung beans (vitexin and isovitexin) significantly attenuated ethanol-induced liver damage by balancing several related biomarkers and improving antioxidant status.

### 3.5. Tea and Coffee

Tea is considered as one of three major beverages along with coffee and cocoa. The cultivation and use of tea trees in China date back to 3000 years ago. Tea is frequently drunk in China as well in other countries. Previously, many studies have reported various benefits of tea, such as anti-cancer, antioxidant, regulation of lipid metabolism, bacteriostasis, preventing cardiovascular disease, and hepatoprotection [198].

Green tea was reported to be used for the treatment of alcohol-induced liver injury. Briefly, green tea protects against alcohol-induced liver injury mainly through an OS mechanism [199]. Another study conducted by Chen et al. [200] also demonstrated that green tea extract ameliorated alcohol-induced liver injury by improving lipogenesis and OS. The results of Lodhi et al. [201] and Park et al. [202] also confirm a hepatic protective effect against alcoholic toxicity. Another mechanistic study reported that green tea attenuated ALD by regulating the PI3K/Akt/eNOS pathway and reducing inflammation in C57BL/6 mice administered with alcohol.

Pu-erh tea is a famous kind of fermented tea produced by fermentation with high humidity and high temperature. During the production process, various microbes (*Saccharomycetes* and *Aspergillus niger*) grow and convert some substances with a unique flavor and aroma in Pu-erh. Pu-erh tea contains high amounts of catechins and polymeric catechins, which show anti-inflammatory, antioxidant, and microbiota-modulating activities. Previously, Wang et al. [203] observed that Pu-erh tea could decrease hepatic histological damage and maintain blood indicators at a normal range in ethanol-induced rats. A recent study conducted by Liu et al. [204] also reported similar results. In detail, Pu-erh tea attenuated OS, lipid accumulation, inflammation, and colon and liver injury by modulating microbiomic and metabolomic responses in ALD. Pu-erh tea could restore the fecal microbiota dysbiosis by increasing the relative abundance of *Allobaculum* and *Bifidobacterium* and reducing the abundance of *Bacteroides* and *Helicobacter*. Furthermore, Pu-erh tea could modulate alcohol-induced metabolomic disorder by regulating purine metabolism, amino acid metabolism (tryptophan and phenylalanine metabolism), and lipid metabolism (glycerophospholipid, sphingolipid, and linoleic acid metabolism). In conclusion, Pu-erh tea is a functional beverage that has strong potential to treat chronic alcohol-associated damage [204].

**Figure 3:**
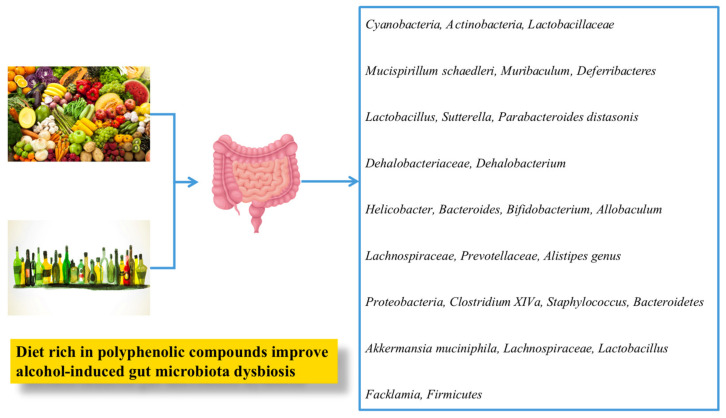
Schematic illustration of effect of edible food plants and bioactive compounds on gut microbiota composition [110,148,158,179,181,190,202,203,204].

Chinese oolong tea, *Litsea coreana*, and cocoa were also reported to attenuate ALD [205,206,207] (Figure 3 and Table 2).

## 4. Conclusions

**Figure 4:**
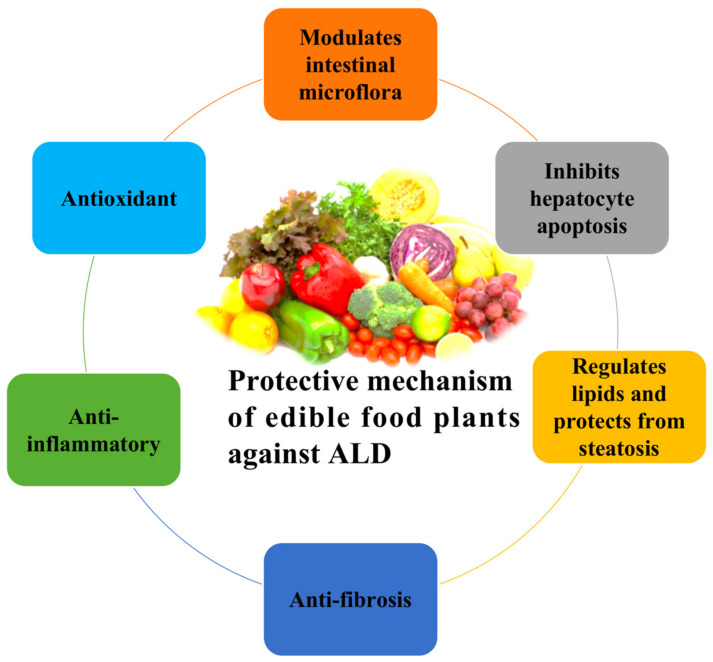
Protective effect of bioactive compounds and edible plant extract from ALD.

ALD is characterized by a broad spectrum of hepatic disorders ranging from simple steatosis/AFL to complicated lesion of liver damage as well as steatohepatitis, cirrhosis/fibrosis, and HCC. A number of studies on ALD have confirmed that liver cell injury, OS, gut microbiota, regeneration, and inflammation play key roles in the progression of this fatal disease and also provide opportunities for the prevention of ALD. In this review, we summarized novel studies (both in vitro and in vivo) for the prevention and treatment of ALD. These results reveal that edible food plants and their derived bioactive compounds exert curative effects on ALD by modulating redox stress, gut microbiota, and anti-inflammation, improving lipid metabolism and anti-proliferation, and promoting autophagy (Figure 4). On the basis of these results, edible food plants and their bioactive compounds have shown to be an ideal candidate for the prevention and treatment of ALD. In the future, mechanistic studies should be carried out on other bioactive compounds and elucidate their mechanisms of action against ALD. Moreover, the possible side effects and effective dosage of these bioactive compounds or extracts in humans need to be further explored.

## Figures and Tables

**Table 1:** Protective effects of dietary polyphenols against ALD.

Categories	Compound Names	Study Design	Key Findings	Ref.
Flavonols	Quercetin	Quercetin (100 mg/kg bw) with ethanol (28% of total calories as ethanol) were given to mice for 12 weeks	Quercetin ameliorated liver injury mediated by chronic-plus-binge ethanol by decreasing ALT and AST, decreased PLIN2 level, activated AMPK activity, and increased co-localization of liver LC3II and PLIN2 proteins. These results predict the regulatory effect of quercetin on lipophagy induced by ethanol.	[69]
	Quercetin, quercetin-3-glucoside, and rutin	HepG2 cells were treated with quercetin, quercetin-3-glucoside, and rutin for 1 h and after that with 5% alcohol for 24 h	Quercetin, quercetin-3-glucoside, and rutin may have preventive strategies against ALD mainly by decreasing hepatic ALT, inflammatory, antioxidant response in HepG2 cells.	[70]
	Quercetin	C57BL/6J mice were fed with regular or ethanol-containing Lieber–DeCarli liquid diets along with quercetin (100 mg/kg bw) for 15 weeks	Quercetin ameliorated lysosomal autophagy dysfunction mediated by ethanol and also exerted autophagic flux suppression, decreased abnormal LC3II and p62 accumulation, elevated LAMP1, LAMP2, and Rab7 expression, and regulated mTOR-TFEB pathway.	[74]
	Fisetin	C57BL/6J mice were fed Lieber–DeCarli or ethanol diet for 4 weeks with or without fisetin (10 mg/kg/day)	Fisetin downregulated ALT, AST, and hepatic NADPH oxidase 4 levels and improved hepatic antioxidant activities. Moreover, it also attenuated alcohol-induced hepatic steatosis via p-AMPK, CYP4A, ACOX1, and MTTP.	[75]
	Fisetin	Fisetin (5 and 10 mg/kg) and 50% ethanol p.o. (10 mL/kg body weight) were given to animals	Fisetin protected against ALD by improving antioxidant activities and restoring mitochondrial respiratory enzymes and matrix metalloproteinase activities.	[76]
	Dihydromyricetin	Dihydromyricetin at dosage of 5 and 10 mg/kg; intraperitoneal injection and ethanol were given to C57BL/6J mice	Dihydromyricetin reduced liver injury markers and TG, increased activation of AMPK and downstream targets CPT-1 and ACC-1, and reduced the expression of proinflammatory cytokines and chemokines in mice and also in cell models.	[79]
	Morin	Morin (50 mg/kg bw) was administered along with 40% alcohol (40%, 2 mL/100 g/day, p.o.) for 21 days to rats	Morin protected from ALD by decreasing ALT, TB, SGPT, and SGOT and improved antioxidant activities in the ethanol-fed rats.	[80]
	Kaempferol	Mice were intragastrically administered distillate spirits (2, 4, 6, 8 g/kg, 50% alcohol, *v*/*v*) twice daily at 3-day intervals and finally 10 g/kg bw for 28 days. Kaempferol was administered at dosage of 10 and 20 mg/kg of bw for 28 days twice a day	Kaempferol treatment significantly reduced MDA, TG, and CYP2E1 and also increased GSH and SOD levels.	[82]
	Kaempferol	Kaempferol (25, 50, 100 mg/kg bw) and ethanol (5 g/kg) were given to ICR mice	Kaempferol may act as a prophylactic treatment against ALD by increasing the expression of butyrate receptors, transporters, and TJ proteins in the intestinal mucosa, decreasing ALT and AST levels.	[84]
Isoflavones	Genistein and puerarin	Genistein and puerarin (0.3 mM/kg bw) and 50% alcohol were administered 1 h later for 5 weeks to ICR mice	Genistein and puerarin exerted protective effect against ALD by downregulating ALT, AST, hepatic lipids, and inflammation biomarkers.	[85]
	Genistein	Genistein group received genistein (16 mg/kg/day bw dissolved in 50% alcohol) and alcohol (50%, 8 g/kg/day bw) for 4 weeks	Genistein administration decreased serum ALT and hepatic MDA, increased GSH levels, and decreased inflammatory markers (IL-18 and TNF-α).	[86]
Flavones	Luteolin	Mice were fed with chronic (1–4% for 3 day and 5% for 9 day) binge ethanol (30% ethanol) and luteolin (50 mg/kg)	Luteolin treatment decreased ALT, TG, LDL-C, lipid accumulation, SREBP1C, FAS, ACC, and SCD1. Moreover, luteolin abrogated the ethanol-induced reduction of AMPK and SREBP-1c phosphorylation.	[89]
	Wogonin	RAW264.7 cells were treated with 100 mM ethanol for 24 h or incubating with various concentrations of wogonin (1.25 to 20 µg/mL). Wogonin-treated mice at the dose of 25, 50, 100 mg/kg/day and ethanol-fed mice were fed diets containing 5% *v*/*v* ethanol for 16 days, and mice were given single binge ethanol administration (20% ethanol, 5 g/kg, bw,) on last day	Wogonin remarkably attenuated ALD by inflammatory response (TNF-α and IL-6) and suppressed PPARγ-meditated phosphorylation and activation of NF-κB p65.	[90]
	Baicalin	Mice were fed with ethanol Lieber–DeCarli diet for 10 days; after that, a single dose of ethanol (5 g/kg bw) and baicalin was injected i.p. (200 mg/kg/day) for 11 days.	Treatment with baicalin attenuated ethanol-induced oxidative stress, inflammation, and cell death.	[92]
	Baicalin	Human normal liver cell L02 were treated with 100 mM alcohol in the absence/presence of baicalin (25, 50 µM) for 24 h Rats were intragastrically administered alcohol (65% *v*/*v*, 5 mL/kg/day) for 3 days and then 10 mL/kg/day on the following days; baicalin groups intragastrically received baicalin (120 mg/kg/day) for 4 weeks	Baicalin supplementation alleviated ALD by decreasing MDA and the proinflammatory cytokines (TNF-α, IL-1β, and IL-6) expression, increasing SOD and GSH-Px. Moreover, it also modulated Shh pathway activation and upregulated expression of Ptc, Smo, Gli-1, and Shh.	[93]
	Apigenin	Mice were fed with 56% erguotou wine or apigenin (150–300 mg/kg) for 1 month	Apigenin reduced the expression of CYP2E1, NF-κB proteins, MDA, and TNF-α, whereas GSH and GSH-Px were increased. Furthermore, apigenin also increased the expression of PPARα and CPT1A and decreased the expression of SREBP1 and FAS.	[94]
	Chrysin	Ethanol was given to rats (5–12 g/kg bw per week) for 4 weeks and chrysin (20 and 40 mg/kg bw) prior to ethanol administration	Chrysin-treated rats ameliorated ALD by inhibiting the activities of ADH, XO, CYP2E1, and CAT levels, respectively.	[95]
Flavanones	Naringin	Zebrafish larvae were treated with 2% ethanol 32 h and naringin (6.25, 12.50, and 25 mg/L)	Naringin protected from alcohol by attenuating lipid accumulation and reducing oxidative stress and apoptosis.	[96]
	Naringenin	Zebrafish larvae were treated with 350 mM ethanol for 32 h and naringenin (2.5, 5, and 10 mg/L)	Naringenin markedly decreased alcoholic liver morphological phenotypes and expression of alcohol and lipid metabolism (*Fads2*, *Cyp2y3*, *Hmgcra*, *Fabp10a*, *Hmgcrb*, *Echs1*, and Fasn) and attenuated hepatic apoptosis in ethanol-induced zebrafish larvae model.	[97]
	Hesperidin	Zebrafish larvae were treated with 350 mM ethanol 32 h and hesperidin (6.25, 12.50, 25 µg/mL)	Hesperidin inhibited alcoholic injury to liver of zebrafish larvae by reducing the hepatic morphological damage and expressions of alcohol and lipid metabolic genes (*Cyp2y3*, *Hmgcra*, Cyp3a65, *Hmgcrb*, *Fads2*, and *Fasn*) with comparison to ALD model.	[98]
Flavan-3-ols	EGCG	Chronic ethanol administration (6 g/kg/day × 60 days) and EGCG (100 mg/kg/day) were given to rats	EGCG ameliorated protein and lipid damage mediated by ethanol.	[101]
	EGCG	*L*. *plantarum* and EGCG combination beads were administered to rats by oral gavage for 8 weeks	EGCG and *L*. *plantarum* markedly attenuate ALD by targeting various molecular markers TNF-α, NF-kB/p50, IL12/p40, and TLR4.	[102]
	Catechin	Ethanol (35% (*v*/*v*) at a dose of 10 g/kg per day) for 2 weeks followed by 14 g/kg/day for 10 weeks; catechin (50 mg/kg) was co-supplemented after 4 weeks	Catechin supplementation ameliorated ALD by downregulating the endotoxin-mediated activation signaling molecule NF-κB and the downstream signaling cascade NO, ROS, and TNF-α and increased the antioxidant biomarkers.	[103]
	EGCG	EGCG (50 mg/kg) along with ethanol was given to mice	EGCG attenuated ALD in ethanol-fed mice.	[104]
Proanthocyanin and Anthocyanin	Cyanidin-3-glucoside	LX-2 cells were treated with ethanol (50 mM) plus 0.1 mM palmitate and cyanidin-3-glucoside (2 mM) for 72 h. Mice were fed with a high-fat high-cholesterol diet plus ethanol and cyanidin-3-O-β-glucoside (200 mg/kg bw) for 12 weeks.	Cyanidin-3-glucoside supplementation reversed the liver damage induced by alcohol. Moreover, it also restored intracellular energy and increased AMPK phosphorylation and autophagy.	[106]
	Cyanidin-3-glucoside	Cyanidin-3-glucoside (200 mg/kg bw) and ethanol were given to mice for 8 weeks	Cyanidin-3-glucoside suppressed NF-κB acetylation, NLRP3 inflammasome activation, and proinflammatory cytokines release.	[107]
	Oligomeric proanthocyanins	Mouse AML-12 hepatocyte cells treated with alcohol and/or OPC (50 µM) for 24 h Oligomeric proanthocyanins (50 mg/kg bw) along with ethanol were given to mice for 9 days	Oligomeric proanthocyanins significantly improved alcohol-induced dyslipidemia, alleviated liver steatosis, reduced levels of ALT, AST, TG, TC, LDL-C, and MDA, increased SOD and HDL-C levels, and decreased the expressions of lipid synthesis genes (SREBP-1, 2) and inflammation gene (TNF-α, IL-1β, and IL-6).	[113]
Alkaloids	Berberine	Acute alcohol exposure model: berberine pretreated orally (200–300 mg/kg/day) for 10 days and after the last dose of ethanol (6 g/kg) at 12-h intervals to the animals Chronic ethanol exposure mouse model: mice were fed with Lieber–DeCarli liquid diets containing 0% or 36% ethanol and berberine (120 mg/kg/day)	Berberine reduced hepatic lipid peroxidation and GSH, suppressed cytochrome P4502E1, and blunted the lipid accumulation.	[114]
	Berberine	Mice were fed with ethanol (5 g/kg body weight) by gavage at days 11, 22, and 33 and berberine-treated (10–100 mg/kg) by gavage	Berberine protected ALD via modulation of gut microbiota and expansion of immuno-suppressive cells.	[115]
	Nuciferine	HepG2 cells with 3 and 10 µM nuciferine for 24 h Nuciferine (3 and 10 mg/kg bw) were intraperitoneally injected once daily for 7 continuous days, and after that, mice were administered with alcohol (5 g/kg) every 12 h for a total of 3 times	Nuciferine alleviated ALD by modulating miR-144/Nrf2/HO-1 cascade.	[116]
Lignans	Silymarin	Mice were fed with ethanol (5 g/kg bw) gavage every 12 h for a total of 3 doses and silymarin (200 mg/kg bw)	Silymarin protected from ALD by decreasing ALT, lipid peroxidation, and TNF-α and increasing GSH level.	[121]
Stilbenes, Saponins, and Terpenoids	Oleanolic acid	Rats were treated with oleanolic acid (10 mg/kg bw) and ethanol (4 g/kg bw) for 1 month	Oleanolic acid protected rats against ALD via induction of Nrf2-related antioxidant to maintain redox, inflammatory pathway, and by modulating ethanol metabolism.	[135]
	Resveratrol	Rats were fed with alcohol 6% (*v*/*v*) and gradually increased to 20% (*v*/*v*) by the fifth week and resveratrol (250 mg/kg bw)	Resveratrol alleviated ALD via regulation of oxidative stress, inflammation, and apoptosis.	[138]
	Polydatin	Zebrafish larvae at 4 days post-fertilization were exposed to ethanol (350 mmol/L) for 32 h and after that treated with polydatin for 48 h	Polydatin strongly alleviated hepatic steatosis, reduced alcohol and lipid metabolism genes (*Fasn*, *Cyp2y3*, *Cyp3a65*, *Hmgcrb*, and *Hmgcra*), inhibited oxidative stress, and upregulated DNA damage-related genes (*Chop* and *Gadd45aa*) in zebrafish model.	[139]
Tannins	Ellagic acid	HepG2 cells were treated with ethanol and ellagic acid (1, 10, and 100 µM)	Ellagic acid protected ethanol-induced toxicity in HepG2 cells.	[140]
	Geraniin and amariin	Mouse liver slices were treated with ethanol (1.7 M) or geraniin, amariin (0.2 mM) for 2 h at 37 °C	Both ellagitannins (geraniin and amariin) effectively protected mouse liver slices from ethanol-induced cytotoxicity and apoptosis by decreasing oxidative damage and modulating Bax/Bcl-2 ratio.	[141]
	Ellagic acid	Rats were fed with alcohol orally (20%, 7.9 g/kg bw) for 45 days and treated with ellagic acid (30–90 mg/kg bw) via intragastric intubation	Ellagic acid effectively modulated oxidative stress, improved antioxidant status, and decreased NO, hydroperoxides PCC, and TBARS in rats.	[142,143]

bw, body weight.

**Table 2:** Protective effects of various edible food plants against ALD.

Edible Food Plant Category	Source	Bioactive Compounds	Study Design	Major Findings	Ref.
Fruits	Blueberry	ND	Blueberry juice combined with mixed probiotics containing *(Bifidobacterium*, *Lactobacillus bulgaricus*, and *Streptococcus thermophilus*; blueberry juice: 1.5 mL/100 g; 20 mL/100 g probiotics) for 10 days were given to ethanol-induced mice.	Blueberry juice and probiotics increased SOD, GSH, and HDL-C levels, decreased AST, ALT, TG, TC, LDL-C, and MDA, suppressed acetylated FOXO1, FOXO1, FasL, and caspase-3, and increased the SIRT1 in ethanol-exposed mice.	[110]
	Mango	Mangiferin	Mangiferin (50 and 100 mg/kg bw) was orally given to ethanol-exposed rats for 12 weeks.	Mangiferin effectively regulated metal elements and FFA in serum, modulated specific alcohol-hepatitis-related genes, metabolic pathways, and potential biomarkers in alcoholic hepatitis rats.	[144]
	Grape	Quercetin, myricetin, rosmarinic acid, catechin, b-type procyanidin trimer, caffeic acid-O-hexoside, epicatechin	Grape-leaf extract (250–500 mg/kg) was orally given to ethanol-induced rats for 12 days.	Grape leaf extract attenuated liver injury by improving antioxidant activities, suppressed NF-κB p65 and proinflammatory cytokines (TNF-α), and normalized histopathological changes in liver.	[145]
	Pomegranate	ND	Pomegranate (600 mg/kg bw) was orally given to ethanol-induced female Fischer wild-type rats for 10 days.	Pomegranate pretreatment markedly reduced alcohol-mediated plasma endotoxin, gut barrier dysfunction, and inflammatory biomarkers and inhibited elevated oxidative and nitrative stress marker proteins. Moreover, pomegranate also restored the levels of intestinal tight junction proteins (claundin-3, ZO-1, occludin, and claudin-1).	[148]
	Cranberry	Cyanidin 3-O-galactoside, peonidin 3-O-galactoside and peonidin 3-O-arabinoside, (+)-catechin, (−)-epicatechin and (−)-epicatechin 3-gallate, procyanidin oligomers, myricetin aglycone, quercetin derivatives, benzoic acid, hydroxycinnamic acid derivatives, and hydroxybenzoic acids	Male albino Wistar rats were received cranberry polyphenols daily, 4 mg/kg bw, along with 4 g/kg bw for 8 weeks	Cranberry polyphenols ameliorated alcoholic liver damage and hepatic steatosis, decreased TG, AST, and ALT activities, diminished TNF-α, TGF-β levels, and free radical generation in mitochondria during intoxication.	[149]
	Wolfberry	Zeaxanthin dipalmitate	BRL-3A cells were treated with ethanol (250 mM) or Wolfberry-derived zeaxanthin dipalmitate (1 µM). Wolfberry-derived zeaxanthin dipalmitate (10 mg/kg bw) was administered to ethanol-induced rats for 4 weeks.	Wolfberry-derived zeaxanthin dipalmitate attenuated hepatocyte and whole-liver injury in both ethanol-treated cells and rat model. The underlying mechanism was mainly due to Wolfberry-derived zeaxanthin dipalmitate directly targeted on cell membrane and including receptor P2 × 7 and adipoR1 which further modulate PI3K/AMP-FoXO3 pathways to restore mitochondrial autophagy. Moreover, WZD also alleviates hepatic inflammation by suppressing NLRP3 inflammasome.	[150]
	Mango	Mangiferin	Mangiferin (100 and 200 mg/kg bw) was orally given to ethanol-exposed rats for 11 days.	Mangiferin attenuated liver injury induced by chronic plus a single binge ethanol by restoring PDE3B stability, which further activated the AMPK/TBK1 signaling and inhibited NF-κB activation, leading to decreased FFA.	[151]
	Lychee	Procyanidin B2, quercetin, 3-O-rutinoside-7-O-a-L-rhamnosidase, isorhamnetin-3-O-rutinoside, (−)-epicatechin, rutin	Lychee pulp (0.4 to 0.8 g/L) was given to mice along with ethanol-containing liquid diet (4%) for 8 weeks.	Lychee pulp ameliorated ALD by decreasing TG, improved the antioxidant status, reduced Nrf2, suppressed lipid synthesis genes, elevated fatty acid β-oxidation expression, and decreased the serum endotoxin level.	[153]
	Lychee		Lychee pulp (0.2 and 0.4 g/kg bw) was given to mice along with ethanol-containing liquid diet for 8 weeks.	Lychee pulp supplementation decreased ALT and AST levels, inhibited serum and hepatic oxidative stress, suppressed mitochondrial 8-hydroxy-2’-deoxyguanosine level, and elevated the hepatic ATP level, mitochondrial membrane potential, activities of mitochondrial complexes I and IV, and mitochondrial DNA content.	[154]
	Lychee		Lychee pulp (0.2 and 0.4 g/kg bw) was given to mice along with ethanol-containing liquid diet for 8 weeks.	Lychee pulp phenolic extract alleviated ethanol-induced liver injury in treated mice via reversed alteration of intestinal microbiota composition, downregulated inflammation markers, increased the expression of intestinal tight junction proteins, antimicrobial proteins, and mucus protecting proteins, repressed NF-κB p65, and suppressed CD14 and TLR4 expression.	[155]
	Blueberry	ND	Blueberry polyphenols extract (100 and 200 mg/kg bw) was orally given to ethanol-exposed mice for 30 days.	Blueberry polyphenols decreased the TG lipid droplet content in liver and serum TG and TC levels and decreased lipogenic and increased lipodieretic mRNA levels. Blueberry polyphenols promoted autophagy to accelerate lipid metabolism and thus protect from ALD.	[156]
	Mulberry		Water extracts of mulberry (0.3 g/kg bw) were orally administered to chronic ethanol-induced rats.	Water extracts of mulberry decreased TG level and MDA contents, increased glycogen deposits, prevented the disruption of the hepatic cells and nuclei, and decreased Firmicutes to Bacteroidetes ratio.	[158]
	Indian gooseberry	ND	Indian gooseberry was administered (250 mg/kg bw) to alcohol-exposed rats.	Indian gooseberry significantly reduced lipid peroxidation levels and restored antioxidant level.	[160]
	Ginseng berry	Ginsenoside F5, ginsenoside Rd, ginsenoside F3, and ginsenoside Re	Ginseng berry extract at the dosage of 0.5–5 mg/mouse along with ethanol was given to mice for 10 days.	Ginseng berry attenuated ALD by improving antioxidant level and reducing inflammatory mediators.	[161]
	Apricot	3-caffeoylshikimic acid, 3-feruloylquinic acid, 3-hydroxy-3-methoxycarbonyl glutaric acid, 1,5-dimethyl citrate, 3,4,5-trimethoxyphenyl-β-D-glucopyranoside, prunate, methyl 3-caffeoylquinate, 3-O- caffeoylquinic acid	AML-12 cells were treated with ethanol or chlorogenic acid. Apricot extract (100 mg/kg bw) along with alcohol (1 g/kg bw) was orally given to mice for 5 days.	Chlorogenic acid derived from apricot extract ameliorated ALD in AML-12 cells by inhibiting alcohol-induced apoptosis, MAPK activation, and antioxidant activities. Apricot extract protected ALD by suppressing lipogenesis in liver tissue, inhibiting activation of SREBP-1, and suppressing hepatic apoptosis and inflammation via ROS-mediated p53 signaling pathway in mice with alcohol-induced liver injury.	[162]
	Lemon	ND	Lemon juice (10 mL/kg bw) was orally given to alcohol-induced C57BL/6 mice for 15 days.	Lemon juice markedly inhibited alcohol-induced increase of ALT, AST, lipid peroxidation levels, and hepatic TG, improved antioxidant capacity (SOD and CAT), and improved histopathological changes in ALD mice.	[164]
	Citrus depressa	5-O-demethylnobiletin, sinensetin, tangeretin, and nobiletin	Citrus depressa extract (300 mg/kg) was orally administered to ethanol-induced mice for 8 weeks.	Citrus depressa extract remarkably decreased AST, ALT, TNF-α levels, hepatic MDA, and CYP2E1 expression, and increased glutathione in ALD mice.	[165]
	Noni fruit	ND	Noni fruit was orally given to ethanol-exposed mice.	Noni fruit reversed the ethanol-induced changes in mice such as ALT, AST, gamma-glutamyl transferase, LDL-C, HDL-C, TG, and TC.	[166]
Vegetables	Purple potato	Petunidin-3-glucoside, Petunidin-3-rutinoside-5-glucoside, Petunidin-3-caffeoyl-rutinoside-5-glucoside	Purple potato extract was administered at the dosage of 5 and 10 mg/kg bw to ethanol-exposed mice for 5 weeks.	Purple potato extract ameliorated ALD by decreasing ALT, AST, TG, and TC, reducing MDA contents and CYP2E1 protein expression, and increasing GSH and SOD levels in ethanol-exposed mice.	[170]
	Garlic oil	ND	Human normal cell LO2 was treated with ethanol (100 mM). Garlic oil was administered (50 to 200 mg/kg bw) to ethanol-exposed male Kunming mice.	Garlic oil decreased n-SREBP-1c and CYP2E1 and increased PPAR-α protein levels in human normal cell L02. Garlic oil decreased n-SREBP-1c and CYP2E1 and increased PPAR-α protein levels in ethanol-induced mice. Additionally, garlic oil decreased FAS and inhibited ethanol-induced hepatic mitochondrial dysfunction.	[175]
	Asparagus officinalis	ND	Asparagus extracts (400 mg/kg bw) were orally administered to male Wistar rats for 70 connective days.	Edible asparagus protected from toxicity mediated by alcohol by improving antioxidant status.	[178]
	Okra seed oil	Polyunsaturated fatty acids; ROS: reactive oxygen species; short-chain fatty acids; monounsaturated fatty acids	Okra seed oil (400 and 800 mg/kg bw) was given to mice for 8 weeks.	Okra seed oil attenuated alcohol-induced liver damage via inhibition of liver fat accumulation, decreased MDA content, decreased hepatic pro-inflammatory cytokines (IL-6, TNF-α, and IL-1), increased SOD and GSH levels, and attenuated lipid metabolic disorder. Furthermore, okra seed oil also modulated gut microbiota dysbiosis by enhancing the *Bacteroidetes* population and reducing the *Proteobacteria* proportion, *Staphylococcus*, and *Clostridium XlVa*.	[179]
	Artichoke	ND	Ethanolic extract of artichoke (0.4 to 1.6 g/kg) was given to ethanol-induced ICR mice for 10 days.	Artichoke remarkably attenuated ALD by preventing elevated levels of ALT, AST, TG, and TC, increased SOD and GSH, decreased MDA level, and suppressed inflammatory pathway (TLR4/NF-κB) in ethanol-induced ICR mice.	[180]
	Rhubarb	ND	Rhubarb extract (0.3%) was given to C57BL/6J mice for 17 days.	Rhubarb extracts protected alcohol-induced liver injury by modulating intestinal microflora, improving antioxidant level, and reducing inflammatory response.	[181]
	Bitter gourd	ND	Bitter gourd was administered (500 mg/kg bw) to C57BL/6 mice fed an alcohol-containing liquid diet for 30 days.	Bitter gourd supplementation reduced the steatotic alternation of liver histopathology, decreased AST, ALT, hepatic TG level, and MDA content, improved antioxidant defense system (SOD, GSH, GRd, GPx, and CAT), reduced pro-inflammatory cytokine levels (IL-6, TNF-α, and IL-1β), and suppressed ACC, CYP2E1, FAS, and SREBP-1 protein expression in alcohol-induced mice.	[182]
Spices	Cinnamon	ND	Cinnamon bark extract (0.5 mL) was administered for 4 days prior to ethanol, and on 5th day, ethanol (6 g/kg bw) was administered. Murine RAW 264.7 macrophage-like cells were treated with cinnamon bark extract (4 µL).	Cinnamon bark extract protected liver from alcohol via the inhibition of MyD88 expression both in vitro and in vivo.	[183]
	Fenugreek	ND	Fenugreek seed polyphenol extract (200 mg/kg bw) and ethanol (6 g/kg per day) were fed to rats for 30 days.	Fenugreek seed polyphenol extract inhibited lipid accumulation in ethanol-induced rats.	[184]
	*Crocus sativus* L.	Safranal, crocin, myricetin, and quercetin	*Crocus sativus* L. (saffron) petal extract was administered (167.5 and 335 mg/kg/day) to ethanol-induced rats for 30 days.	Saffron polyphenolic extract protected liver from ethanol by reducing inflammation in ethanol-administered rats.	[185]
	Parsley oil	ND	Parsley oil (50 mg/kg bw) was given to adult male albino rats for 4 weeks.	Parsley oil attenuated alcohol-induced liver injury by oxidative stress mechanism.	[186]
	*Syzygium aromaticum L*.	ND	Polyphenol-rich extract of clove buds (Clovinol) (100 mg/kg bw) was given to ethanol-induced rats for 30 days.	Clovinol decreased alcohol-associated oxidative stress and inflammatory changes in ethanol-induced rats.	[187]
	*Thymus vulgaris*	ND	*Thymus vulgaris* leaves were orally given (500 mg/kg bw) to ethanol-induced rats for 21 days.	Co-administration (*Thymus vulgaris* and ethanol) modulated several biomarkers such as ALP, AST, albumin, CAT, MDA, SOD, GST, and lipid profile.	[188]
	Peppers	Capsaicin	Capsaicin was given (10 and 20 mg/kg) to ethanol-induced rats.	Capsaicin ameliorated alcohol-induced liver injury by modulating matrix metalloproteinases and suppressing free radical formation and oxidative stress.	[189]
Cereals	Black rice	Cyanidin-3,5-diglucoside, cyanidin-3-glucoside, cyanidin-3-rutinoside, and peonidin-3-glucoside	Alcohol (3.7 g/kg bw) and anthocyanin-rich black rice extract (125, 250, and 500 mg/kg bw) dissolved in water was administered using an intragastric tube for 45 days.	Anthocyanin-rich black rice extract attenuated ALD by decreasing serum AST, ALT, TCH, TG, and GGT levels and improving antioxidant levels.	[105]
	Rice	Acacetin, caffeic acid, ferulic acid, sinapic acid, p-coumaric acid, quercitrin, vitexin, rutin, hesperidin, ethyl caffeate, and ethyl coumarate	Rice bran phenolic extract (0.25 or 0.50 g/L) was fed along with alcohol-containing liquid diet (4%) to mice for 8 weeks.	Anthocyanin-rich black rice extract supplementation ameliorated ALD by repressing inflammatory responses in liver, intestinal microbiota dysbiosis, and barrier dysfunction and inactivated the endotoxin-TLR4-NF-κB pathway.	[190]
	Rice	Acacetin, caffeic acid, ferulic acid, sinapic acid, p-coumaric acid, quercitrin, vitexin, rutin, hesperidin, ethyl caffeate, and ethyl coumarate	Rice bran phenolic extract (0.25 or 0.50 g/L) was fed along with alcohol-containing liquid diet (4%) to mice for 8 weeks.	Rice bran phenolic extract exerted protective effect against ALD in mice fed with an ethanol-containing diet via microRNAs-PGC-1α-TFAM signal pathway.	[191]
	Tartary buckwheat	ND	Acute liver injury model group: buckwheat ethanol extracts (8.35, 16.70 and 41.75 mL/kg bw) and ethanol (4 g/kg bw) were intragastrically administered to rats for 7 consecutive days. Chronic alcoholic liver injury: buckwheat ethanol extracts (8.35, 16.70 and 41.75 mL/kg bw) and ethanol (3 g/kg/day bw; 37.5% volume fraction) intragastrically administered to SD rats for 8–9 consecutive weeks.	Tartary buckwheat extract administration significantly decreased serum ALT, AST, and hepatic MDA and improved hepatic GSH level.	[192]
	Mung bean extract	Vitexin and isovitexin	Mung bean extract (containing 15 mg vitexin and 13 mg isovitexin, respectively, per kg bw) was given along with spirit (56% alcohol, 16 mL/kg bw) 2 h after the doses of mung bean extract for 14 days.	Mung bean extract decreased ALT and AST and improved antioxidant levels.	[197]
Tea	Pu-erh tea	Gallocatechin, gallic acid, and caffeine	Pu-erh tea extract (1 or 4 g/L *w*/*v* added into drinking water) and ethanol solution (10% *w*/*v*) were administered by gavage for 30 days.	Pu-erh tea extract contributed to the protective effect against ALD by improving oxidative stress, reducing lipid accumulation, reducing inflammation, and modulating microbiomic and metabolomic responses.	[204]

ND, not determined. bw, body weight.
